# H3K79 methylation: a new conserved mark that accompanies H4 hyperacetylation prior to histone-to-protamine transition in *Drosophila* and rat

**DOI:** 10.1242/bio.20147302

**Published:** 2014-05-02

**Authors:** Christine Dottermusch-Heidel, Stefanie M. K. Gärtner, Isabel Tegeder, Christina Rathke, Bridlin Barckmann, Marek Bartkuhn, Sudhanshu Bhushan, Klaus Steger, Andreas Meinhardt, Renate Renkawitz-Pohl

**Affiliations:** 1 Fachbereich Biologie, Entwicklungsbiologie, Philipps-Universität Marburg, Karl-von-Frisch Strasse 8, 35043 Marburg, Germany; 2 Department of Genetics, Justus Liebig University Giessen, Heinrich-Buff-Ring 58-62, 35392 Giessen, Germany; 3 Unit of Reproductive Biology, Department of Anatomy and Cell Biology, Justus Liebig University Giessen, Aulweg 123, 35385 Giessen, Germany; 4 Molecular Andrology Section, Department of Urology, Pediatric Urology and Andrology, Justus Liebig University Giessen, Schubertstrasse 81, 35392 Giessen, Germany; * Present address: Institut de Génétique Humaine, CNRS UPR 1142, 141 Rue de la Cardonille, 34396, Montpellier Cedex 5, France.

**Keywords:** Gpp, Grappa, Gpp-D, Spermiogenesis, Histone-to-protamine transition, H4 acetylation

## Abstract

During spermiogenesis, haploid spermatids undergo extensive chromatin remodeling events in which histones are successively replaced by more basic protamines to generate highly compacted chromatin. Here we show for the first time that H3K79 methylation is a conserved feature preceding the histone-to-protamine transition in *Drosophila melanogaster* and rat. During *Drosophila* spermatogenesis, the Dot1-like methyltransferase Grappa (Gpp) is primarily expressed in canoe stage nuclei. The corresponding H3K79 methylation is a histone modification that precedes the histone-to-protamine transition and correlates with histone H4 hyperacetylation. When acetylation was inhibited in cultured *Drosophila* testes, nuclei were smaller and chromatin was compact, Gpp was little synthesized, H3K79 methylation was strongly reduced, and protamines were not synthesized. The Gpp isoform Gpp-D has a unique C-terminus, and Gpp is essential for full fertility. In rat, H3K79 methylation also correlates with H4 hyperacetylation but not with active RNA polymerase II, which might point towards a conserved function in chromatin remodeling during the histone-to-protamine transition in both *Drosophila* and rat.

## INTRODUCTION

During spermatogenesis, the transition from a nucleosomal histone-based structure to a protamine-based structure is a highly conserved, unique event in most invertebrates and vertebrates, including *Drosophila* and humans (reviewed by Barckmann et al., 2013; Braun, 2001; Oliva, 2006; Rathke et al., 2014). In the haploid phase of mammalian spermatogenesis, called spermiogenesis, somatic histones that build the nucleosomal structure are first replaced by testis-specific histone variants. These histone variants are replaced by small transition proteins, which in turn are replaced by highly basic and much smaller protamines, resulting in highly compacted chromatin with a doughnut-like structure (Braun, 2001; Kimmins and Sassone-Corsi, 2005; Sassone-Corsi, 2002). It is generally accepted that correct protamine loading is a prerequisite for the generation of competent spermatozoa and thus essential for full fertility in mammals, including humans (Baarends et al., 1999; Cho et al., 2001; Prakash, 1989; Steger et al., 2003). Analogous to the situation in mammals, also histones in *Drosophila* are replaced stepwise by transition-like proteins and protamines (Jayaramaiah Raja and Renkawitz-Pohl, 2005; Rathke et al., 2007; Rathke et al., 2010).

The assembly of protamine-based chromatin in *Drosophila* depends on the histone chaperone CAF1 (Doyen et al., 2013; Rathke et al., 2014). It has long been postulated that protamines are needed to protect the paternal genome from mutagens (Chen and McKearin, 2003; Oliva, 2006). In support of this hypothesis, *Drosophila* loss-of-function mutants for the two protamine genes are 20-fold more sensitive to X-radiation (Rathke et al., 2010).

However, to date little is known about how the histone-to-protamine transition is regulated at the molecular level, although some conserved characteristic features accompanying the transition process in mammals and *Drosophila* have been described (for reviews, see Baarends et al., 1999; Braun, 2001; Carrell et al., 2007; Chen and McKearin, 2003; Rathke et al., 2007; Sassone-Corsi, 2002). The replacement process is marked by an increase in hyperacetylated histone H4 just prior to histone displacement and DNA strand breaks during the transition process (Grootegoed et al., 1998; Hazzouri et al., 2000; Leduc et al., 2008). Histone H4 hyperacetylation was therefore believed to act as a starting signal for histone detachment and to trigger the subsequent transition processes. In accordance with this hypothesis, a decrease in histone H4 hyperacetylation correlates with impaired spermatogenesis in mice, humans, and *Drosophila* (Awe and Renkawitz-Pohl, 2010; Fenic et al., 2008; Sonnack et al., 2002). *Drosophila in vitro* culture studies with cysts containing synchronously developing spermatids have demonstrated that inhibition of histone acetylation blocks the progression from a histone-based to a protamine-based configuration, whereas premature hyperacetylation does not lead to a premature histone-to-protamine transition. This led to the conclusion that histone H4 hyperacetylation is essential but is not the sole inducer of the switch from histones to protamines during spermiogenesis (Awe and Renkawitz-Pohl, 2010). Indeed, it has recently been proposed that the H2B histone variant TH2B controls the histone-to-protamine transition in mice (Montellier et al., 2013).

In our study reported here, we searched for putative chromatin-relevant features conserved between *Drosophila* and mammals, specifically the rat, and took advantage of the experimental accessibility of *Drosophila*. We found that the H3K79 methyltransferase Grappa (Gpp) is expressed in canoe stage nuclei during spermiogenesis and that H3K79 methylation is a conserved histone modification that precedes histone removal both in *Drosophila* and rat. In *Drosophila*, both H3K79 methylation in spermatids and chromatin localization or synthesis of the corresponding methyltransferase Gpp were dependent on prior histone acetylation. In rats, both H3K79 methylation and H4 hyperacetylation seemed to be unrelated to active transcription in spermatids, but may fulfill a function in preparing the chromatin for the replacement of histones by protamines.

## MATERIALS AND METHODS

### *Drosophila* strains

*Drosophila* flies were maintained on standard medium at 18°C or 25°C. *w^1118^* (Bloomington Drosophila Stock Center, BL6328) was used as the wild-type strain. The fly strain expressing protB-eGFP (protB, protamine B) was previously generated (Jayaramaiah Raja and Renkawitz-Pohl, 2005). For the generation of protB-mCherry transgenic flies, the same upstream regulatory region and coding sequence as for protB-eGFP were cloned in the transformation vector p*ChabΔSalΔLacZ* in-frame to mCherry, and the recombinant plasmid was injected into *w^1^* embryos (Klemenz et al., 1987) as described previously (Michiels et al., 1993).

The UAS-RNAi construct v110264 was obtained from Vienna Drosophila RNAi Center (VDRC), and the UAS-RNAi constructs HMS00160 (BL34842), JF01284 (BL31327) and JF01283 (BL31481) were obtained from the Bloomington Drosophila Stock Center. RNAi-Constructs are directed against all *gpp* transcripts. The c135-Gal4 (*w^1118^*; P{GawB)c135) driver line should drive expression of UAS constructs in spermatocytes (Hrdlicka et al., 2002) and was obtained from the Bloomington Drosophila Stock Center (BL6978). Transcription of RNAi in spermatogonia and spermatocytes was induced by mating virgins carrying two copies of the Bam-Gal4-VP16 (Chen and McKearin, 2003) driver to males carrying the UAS constructs. Flies were kept at 30°C throughout the experiment.

The hypomorphic semi-lethal *grappa* (*gpp*) allele *gpp^72A^* and lethal allele *gpp^61A^* are described (Shanower et al., 2005). The deficiency spanning the *gpp* gene (*w^1118^*; *Df(3R)Bsc193*/*TM6B*, *Tb^+^*; BL9620) was obtained from the Bloomington Drosophila Stock Center.

### *In situ* hybridization

Whole mounts of adult *Drosophila* testes were hybridized *in situ* according to Morris et al. (Morris et al., 2009) with minor modifications, i.e. re-hybridization, hybridization, and washes in hybridization buffer at 55°C instead of 65°C. DIG-labeled RNA probes used in the hybridizations were generated by *in vitro* transcription of regions of interest using the DIG RNA Labeling Kit (Roche, Germany). These regions, consisting of fragments of 300 to 800 bp of selected regions of the *gpp* gene, were first amplified by PCR from genomic DNA and then cloned into the *pCR®II-TOPO®* Vector (Invitrogen). The following primers were used for amplification: *Gpp-for* 5′-ACTGTTCGCACCACACGTGA-3′ and *Gpp-rev* 5′-GCAGAGCTTCTAGTCCAACA-3′; *gpp-BCE*-*for* 5′-AACGATTTGGCAACGCAACG-3′ and *gpp-BCE-rev* 5′-GGTTGTTTCTGATTTGAAATCTT-3′; *gpp-DE-for* 5′-TGATGAGACCCACTGGCAG-3′ and *gpp-DE-rev* 5′-CTTAAGGGAGCTACCAGCAT-3′; *gpp-E-for* 5′-CAGCTCGCGTGTAGAAAGAT-3′ and *gpp-E-rev* 5′-TTTAGCTCCCACACTGCTTG-3′; *gpp-F-for* 5′-ACTGACCAGGGTATCTGTA-3′ and *gpp-f-rev* 5′-GACTACAAGTGTTACGGGCA-3′.

### Sterility tests

For each genotype, one freshly hatched adult male was placed with two wild-type virgin females in a vial for 5 days at 25°C (*n* = 20 for each genotype). After 5 days, the parental generation was removed from the vials. After 2 weeks, offspring in each vial were counted.

### Immunofluorescence staining of squashed testes

The following antibodies were used in immunofluorescence staining of squashed testes treated essentially as described previously (Hime et al., 1996). To analyze the methylation of histone H3K79 during spermatogenesis, we used polyclonal rabbit anti-dimethyl H3K79 (ab3594; Abcam, Cambridge, UK; 1:1000; slight cross-reactivity with histone H3 monomethyl K79 and trimethyl K79 reported) and rabbit anti-trimethyl H3K79 (ab2621; Abcam; 1:1000; cross-reactivity with histone H3 dimethyl K79 reported). Cross-reactivity between species is strongly expected. For analyzing the acetylation status of histone H4, we used a rabbit polyclonal anti-histone H4 acetyl-antibody (Millipore 06-598; 1:500) that recognizes histone H4 acetylated at lysines 5, 8, 12, and 16. Anti-histone antibody (Millipore MABE71; 1:1200) was used to detect core histones.

For studying expression of Gpp, we raised a rabbit polyclonal peptide antibody (amino acids 1566–1584) that recognized all Gpp isoforms (anti-Gpp-all). The antibody was affinity purified and applied at a dilution of 1:1000 (Pineda Antikörper-Service; http://www.pineda-abservice.de). To determine the specificity of the α-Gpp-all antibody, we performed immunizing peptide-blocking experiments. For this, the antibody was incubated with an excess of the peptide (5–20 µg/ml). The neutralized antibody was compared to the antibody alone in immunofluorescence stainings (supplementary material Fig. S4).

To visualize IgG antibodies, we used Cy2-conjugated (Dianova, 1:40), Cy3-conjugated (Dianova, 1:100), or Cy5-conjugated (Dianova, 1:100) secondary antibodies. Hoechst staining was used to visualize the chromatin. Squashed testes were embedded in Fluoromount-G (Southern Biotech, Birmingham, AL, USA). Immunofluorescence, eGFP, and mCherry signals were examined using a Zeiss Axioplan 2 microscope equipped with appropriate fluorescence filters. Images were acquired with a Zeiss AxioCam MR^m^ digital camera.

### Culture of pupal testes and treatment with inhibitors

Pupal testes (24 h after puparium formation) were dissected, cultured, and treated as described previously (Awe and Renkawitz-Pohl, 2010; Leser et al., 2012). Briefly, pupal testes were dissected in Shields and Sang M3 insect culture medium (Sigma–Aldrich cat. no. S8398) supplemented with 10% fetal bovine serum (heat inactivated, insect culture tested, Sigma–Aldrich cat. no. F3018), 100 U/ml penicillin, and 100 mg/ml streptomycin (Gibco–Invitrogen cat. no. 15140-148). For inhibitor treatment, generally six pupal testes were used for each inhibitor and control per experiment. The experiments were repeated at least three times.

Testes were treated with anacardic acid (Merck Biosciences, cat. no. 172050; 28.69 mM DMSO stock solution) and trichostatin A (Cell Signalling Tech., cat. no. 9950; 4 mM ethanol stock solution) appropriately diluted with culture medium. Control cultures with solvent alone were analyzed in parallel. Cultures were incubated at 25°C for 24 h prior to fixation.

For inhibition of H3K79 methylation, pupal testes were incubated with the Dot1l inhibitor EPZ004777 (Daigle et al., 2011; Epizyme Inc., Cambridge, MA, USA; 1 mM stock in DMSO) at the appropriate dilution (50 µM) in culture medium. Control cultures with solvent alone were analyzed in parallel. Cultures were incubated at 25°C for 24 or 48 h prior to fixation.

### Rat testes sections and immunohistochemical analysis

Sections (4–5 µm) from rat testes were immunohistochemically analyzed according to standard protocols (Bergmann and Kliesch, 1994; Kliesch et al., 1998) with minor modifications. Sections were incubated with primary antibody overnight at 4°C, and then for 1 h at room temperature with biotinylated secondary antibody (Dianova; 1:250), followed by incubation with avidin–biotin complex (Vectastain ABC Elite Kit, Vector Labs, Burlingame, CA, USA) for 45 min with 3,3′-diaminobenzidine as chromogen. The primary antibodies used were: rabbit polyclonal anti-trimethyl-H3K79 (ab2621; Abcam; 1:1000); rabbit polyclonal anti-acetyl-histone H4 antibody (Millipore 06-598; 1:500); or rabbit polyclonal to active RNA polymerase II (CTD repeat YSPTSPS, phospho S5; ab5131; Abcam; 1:500). Sections were analyzed using a Zeiss Axioplan light microscope equipped with a Zeiss AxioCam MR^m^ digital camera.

This study was carried out in strict accordance with the recommendations in the Guide for the Care and Use of Laboratory Animals of the German Animal Welfare Act and local regulations at JLU Giessen.

## RESULTS

### H3K79 methylation precedes the histone-to-protamine switch in *Drosophila*

To identify new regulators of chromatin remodeling involved in the switch from a histone-based chromatin to a protamine-based configuration, we analyzed postmeiotic-enriched transcripts of stage-specific testes transcriptome data published by Vibranovski et al. (Vibranovski et al., 2009) with a particular focus on genes conserved in mammals. In doing so, we identified the gene *grappa* (*gpp*) encoding the histone methyltransferase (HMT) Grappa (Gpp), which has a strong sequence similarity to the family of Dot1-like HMTs. These HMTs particularly exhibit intrinsic methyltransferase activity towards lysine 79 of histone H3 in yeast (Lacoste et al., 2002; Ng et al., 2002), mammals (Feng et al., 2002), and *Drosophila* (Shanower et al., 2005). In *gpp^X^* mutants of *Drosophila*, H3K79 methylation is lacking, as shown by in immunofluorescence and Western blots (Shanower et al., 2005).

**Fig. 1.**
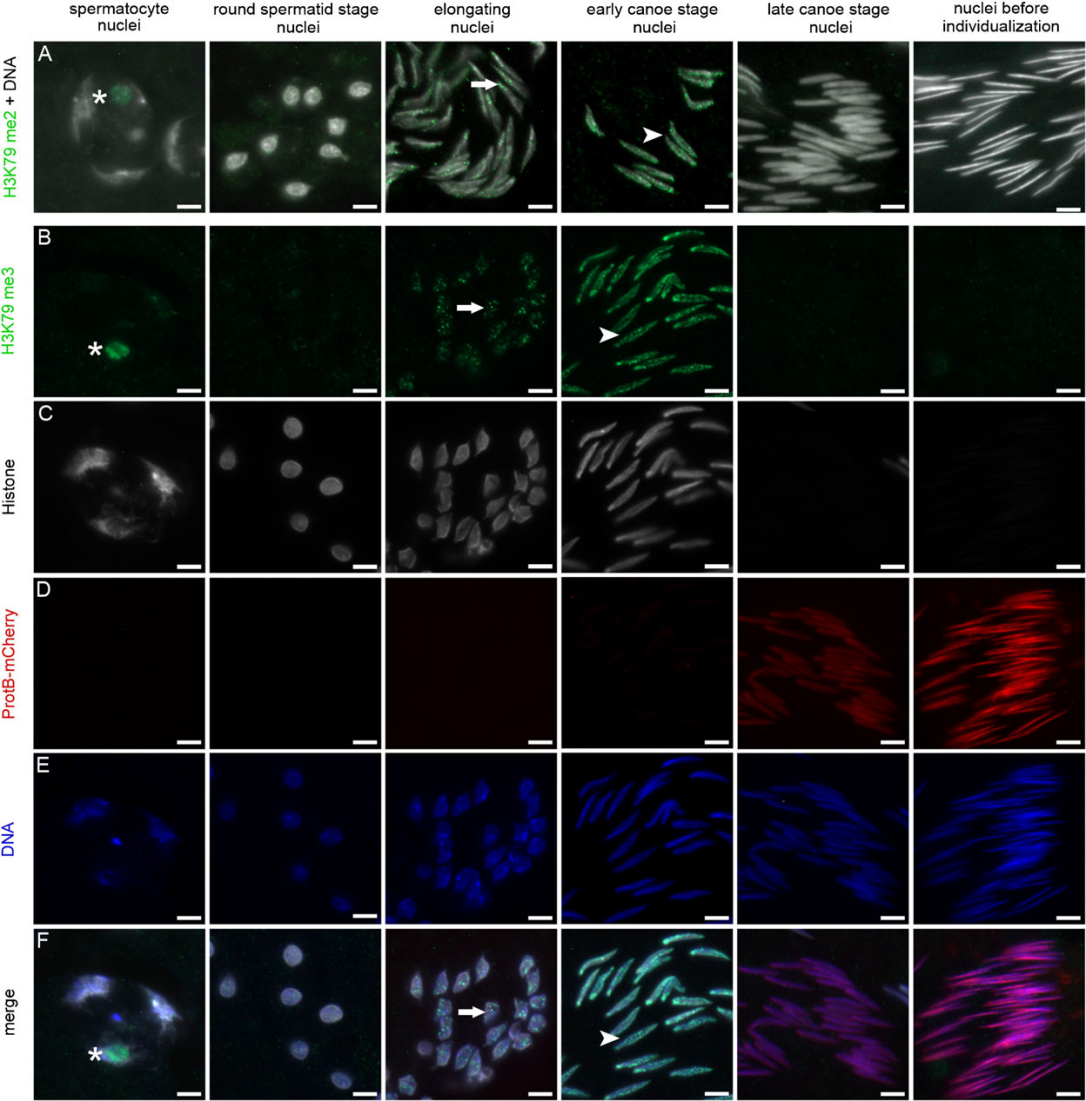
H3K79 methylation precedes the histone-to-protamine switch. (A) Anti-H3K79me2, (B) anti-H3K79me3, and (C) anti-histone staining of squashed spermatid nuclei from testes of *protB-mcherry* flies. (A,E) DNA was visualized by Hoechst staining. (A) H3K79me2 as well as (B) H3K79me3 were present in the nucleolus of spermatocytes (*) and reappeared in the nuclei of elongating spermatids (arrow), reaching a maximum level in early canoe stage nuclei (arrowhead), shortly before histone removal. In stages where protamines are present (D, columns 5 and 6), H3K79me2 and H3K79me3 were no longer detectable (A and B, respectively, columns 5 and 6). (F) Merged image of panels B–E. Scale bars: 5 µm.

Since Gpp and its homologues are the only HMTs known to mediate H3K79 mono-, di-, and trimethylation (Min et al., 2003; Nguyen and Zhang, 2011), we first immunohistologically analyzed the methylation status of lysine 79 of histone H3 during spermatogenesis using squashed nuclei from wild-type testes. Interestingly, both histone H3 dimethylated at position 79 (H3K79me2) and histone H3 trimethylated at position 79 (H3K79me3) were highly dynamically and similarly distributed in male germ cells (Fig. 1A,B, respectively, column 1, asterisk; column 3, arrow; column 4, arrowhead). After meiosis, H3K79me2 and H3K79me3 were present in the nuclei of spermatids starting to elongate (Fig. 1A,B, respectively, column 3, arrow). The strongest signal of H3K79me2 and H3K79me3 was detected in the nuclei of early canoe stage spermatids (Fig. 1A,B, respectively, and merged in F, column 4, arrowhead), when histones were still present (Fig. 1C, column 4). Shortly thereafter, H3K79 di- and trimethylation rapidly vanished (Fig. 1A,B, respectively, columns 5 and 6), when protamine expression commenced in the late canoe stage, depicted by the expression of protB-mCherry fusion proteins (Fig. 1D, columns 5 and 6). As H3K79me2 and H3K79me3 showed a similar distribution in male germ cells, we focused only on H3K79me3 to elucidate the role of Gpp.

### Gpp is essential for full male fertility in *Drosophila*

As is also the case in other organisms, Gpp is the only predicted methyltransferase in *Drosophila* capable of catalyzing mono-, di-, and trimethylation of H3K79 in a non-processive manner (Min et al., 2003; Nguyen and Zhang, 2011; Shanower et al., 2005). Consequently, we aimed at analyzing whether Gpp is required for spermiogenesis and thus for male fertility. However, previous studies have clearly demonstrated that Gpp is already active during embryogenesis. Hence, complete loss-of-function mutants or ubiquitous knockdown of *gpp* results in early larval lethality (Mohan et al., 2010; Shanower et al., 2005), which hinders the investigation of spermatogenesis. Since we did not achieve a germ-cell-specific knockdown of Gpp function with an inhibitor (supplementary material Figs S1, S2) or with RNAi (supplementary material Fig. S3), we analyzed spermiogenesis in testes from males with hypomorphic *gpp* alleles that reach adulthood. For this, we used the hypomorphic, homozygous viable allele *gpp^72A^* in *trans* to the homozygous lethal allele *gpp^61A^*, as well as the homozygous lethal deficiency *Df(3R)Bsc193*. We asked whether the hypomorphic allele of *gpp* has an effect on the efficiency of sperm production or their capacity for fertilization and addressed this question in sterility tests.

**Fig. 2.**
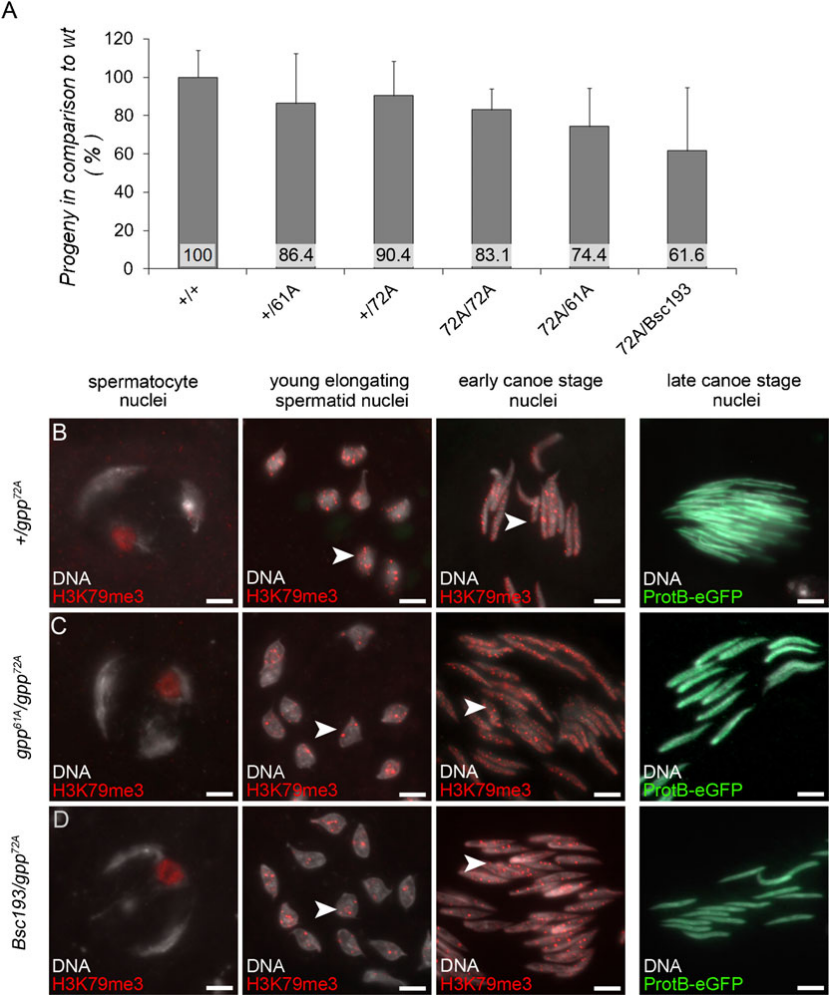
Hypomorph *gpp* mutants show reduced fertility. (A) Sterility tests showing a reduced number of progeny in transheterozygous *gpp* mutants *gpp^72A^*/*gpp^61A^* and in *gpp^72A^*/*Bsc193* males. (B–D) Anti-H3K79me3 staining of squashed spermatid nuclei from testes of *protB-mcherry* flies. DNA was visualized by Hoechst staining. Arrowheads indicate H3K79me3 in nuclei. Neither *gpp^61A^*/*gpp^72A^* mutants (C) nor *Bsc193*/*gpp^72A^* mutants (D) showed a severe decrease in H3K79me3 compared to the control (+/*gpp^72A^*, B). No defects in ProtB-eGFP expression were visible. Scale bars: 5 µm.

Indeed, fertility of transheterozygous males was reduced most strongly in *gpp^72A^/Bsc193*. In this case, fertility was approximately 40% lower than that of control animals (Fig. 2A). These results hint at a function of H3K79 methylation in the generation of fertile sperm. Then we tested whether H3K79 methylation was abolished in these transheterozygous males. Immunohistological staining of squashed spermatid nuclei from testes from transheterozygous combinations of *gpp* alleles detected remaining H3K79me3 in the nuclei of elongating and early canoe stage spermatids (*gpp^61A^*/*gpp^72A^* Fig. 2C and *Bsc193*/*gpp^72A^* Fig. 2D, columns 2 and 3, arrowheads), similar to the control (+/*gpp^72A^* Fig. 2B, columns 2 and 3, arrowheads). Not surprisingly, also protB-eGFP-expressing cysts were visible (Fig. 2C,D, column 4), as in the control (Fig. 2B, column 4), and we detected individualized sperm in the seminal vesicles.

### The H3K79 methyltransferase Gpp is expressed in canoe stage spermatids

**Fig. 3.**
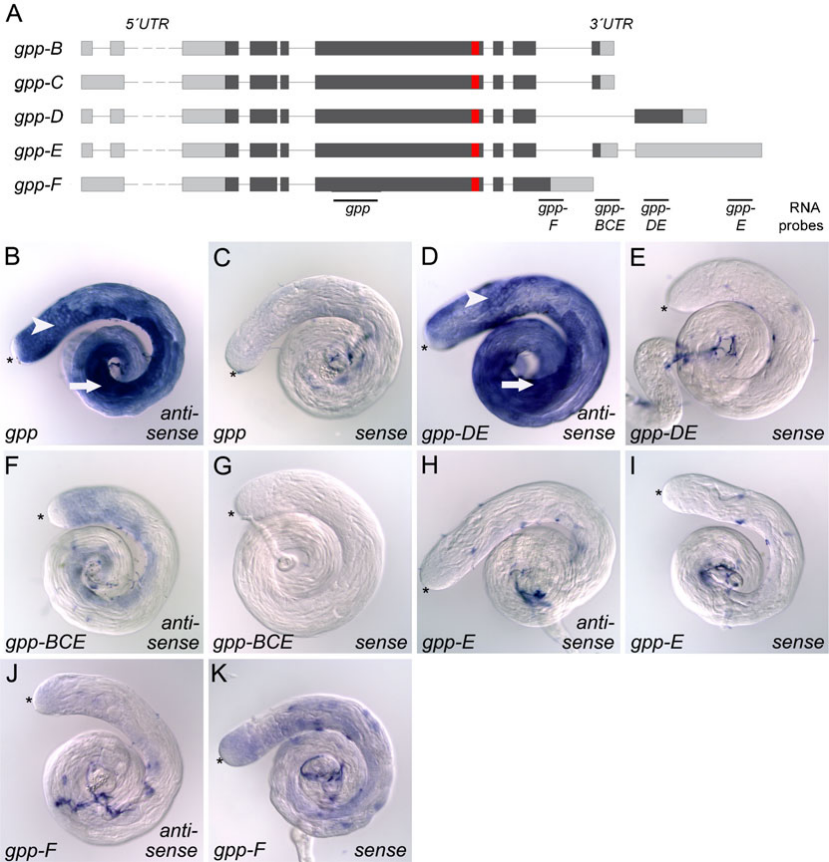
*gpp-D* is the spermiogenesis-relevant isoform. (A) Schematic representation of the *gpp* gene structure. Exons are depicted as boxes, protein-coding regions as dark gray boxes, and introns as thin lines. *gpp* encodes for at least five isoforms that differ in 5′ UTRs and in 3′ regions. Of note, Gpp-D and Gpp-F are characterized by unique C-termini. RNA probes used are shown as dark gray lines (*gpp*, *gpp-DE*, *gpp-BCE*, *gpp-F*, and *gpp-E*). Red boxes are indicating the region of the Gpp antibody recognition sequence. (B–K) *in situ* hybridizations in wild-type testes. (B) With an RNA probe that recognizes the transcripts encoding all Gpp isoforms, a *gpp* transcript was detected from the spermatocyte stage (arrowhead) onwards until spermatids started to elongate (arrow). The hub region (*) and spermatogonia were free of staining. A comparable transcription pattern was visible with a probe that detected the transcripts encoding isoforms Gpp-D and Gpp-E (D; *gpp-D* and *gpp-E*), but not with RNA probes that detected the transcripts encoding Gpp-B, Gpp-C, and Gpp-E (F; *gpp-B*, *gpp-C*, and *gpp-E*), Gpp-E (H; *gpp-E*), or Gpp-F (J; *gpp-F*). (C,E,G,I,K) Hybridizations with sense probes did not yield a signal.

We then studied *gpp* expression during spermatogenesis. The *gpp* gene is characterized by a complex exon–intron structure that encodes at least five different transcripts (*gpp-B*, *gpp-C*, *gpp-D*, *gpp-E*, and *gpp-F*) that differ particularly in the 3′ and 5′ regions (according to FlyBase Release 5.47; Fig. 3A). To analyze which transcripts are present in the testis, we performed *in situ* hybridizations with specific RNA probes. When we used an RNA probe that recognizes sequences common in all isoforms, *gpp* transcripts were specifically detected in spermatocytes (Fig. 3B, arrowhead) and early spermatids (Fig. 3B, arrow). Then we used RNA probes that recognize the transcripts encoding specific Gpp isoforms (Fig. 3A). A transcription pattern comparable to that obtained with the probe that recognized parts of the transcripts present in all isoforms was only seen with an RNA probe directed against transcripts of isoforms Gpp-D and Gpp-E (Fig. 3D; *gpp-D* and *gpp-E*), but not with RNA probes directed against transcripts of isoforms Gpp-B, Gpp-C, and Gpp-E (Fig. 3F; *gpp-B*, *gpp-C*, and *gpp-E*), Gpp-E alone (Fig. 3H; *gpp-E*), or Gpp-F alone (Fig. 3J; *gpp-F*). These results strongly indicated that mainly the transcript encoding the Gpp-D isoform is made in male germ cells. Importantly, this isoform transcript not only differs in the 3′ untranslated region (UTR), but also encodes a longer protein that differs in the C-terminal 344 amino acids.

**Fig. 4.**
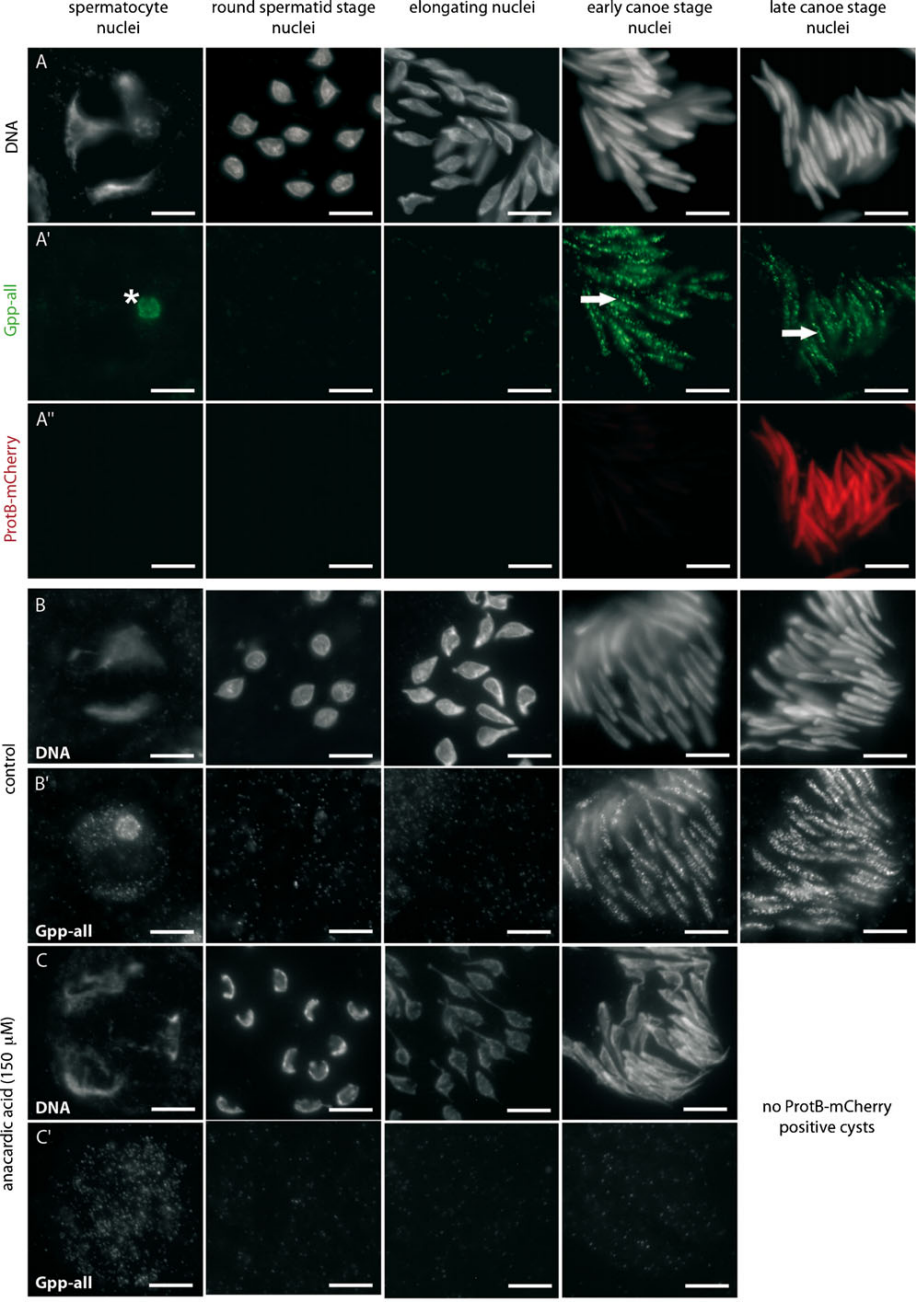
Gpp expression accompanies the histone-to-protamine transition and depends on H4 hyperacetylation. (A–A″) Staining of squashed spermatid nuclei from testes of *protB-mcherry* flies. (A) DNA was visualized by Hoechst staining. (A′) Gpp was distributed in the nucleolus of spermatocytes (*) and mainly localized in a dotted pattern in early and late canoe stage nuclei (arrows). (A″) ProtB-mCherry expression. (B–C′) Squashed preparations of spermatid nuclei derived from cultured pupal testes of *protB*-*mcherry* flies incubated for 24 h with either detergent (control; B,B′) or the acetyltransferase inhibitor anacardic acid (C,C′). Gpp was detected by staining with anti-Gpp-all (B′,C′). DNA was visualized by Hoechst staining (B,C). After treatment with anacardic acid, Gpp was not detected at the early canoe stage (compare B′ to C′). Scale bars: 10 µm.

A characteristic feature of *Drosophila* spermiogenesis is that many proteins are stage-specifically translated from stored silent mRNAs synthesized before meiotic divisions (for a review, see Renkawitz-Pohl et al., 2005). Therefore, we aimed at analyzing Gpp distribution. We raised an antibody against a peptide of Gpp (amino acids 1566–1584) and proved the specificity of the antibody in a peptide-blocking assay (supplementary material Fig. S4). Immunostainings with an antibody that recognizes all Gpp isoforms revealed a low expression level from the chromosomes of spermatocytes and significant staining of the nucleolus (Fig. 4A′, details of spermatocyte expression are shown in supplementary material Fig. S5). In the following, we focused on stages after meiosis. After meiosis, we observed a dotted distribution of the methyltransferase in early and late canoe stage nuclei (Fig. 4A′, arrows); late canoe stage is the stage in which histones are replaced by protamines (Fig. 4A″). We observed a lower level of H3K79 methylation already a bit earlier, as the nuclei elongated (Fig. 1B), which might be due to a low but not yet detectable level of Gpp.

### Full level of H3K79 methylation depends on histone hyperacetylation

As the pattern of H3K79me3 (Fig. 1B) strongly resembles that of H4 hyperacetylation in elongating and early canoe stage spermatids, we next asked whether H3K79 methylation is dependent on histone acetylation in spermatids. To test this hypothesis, we used the testes culture system developed in our laboratory to inhibit histone acetylation or promote premature histone acetylation by using specific inhibitors (Awe and Renkawitz-Pohl, 2010) and analyzed the methylation status of H3K79me3 in treated testes.

**Fig. 5.**
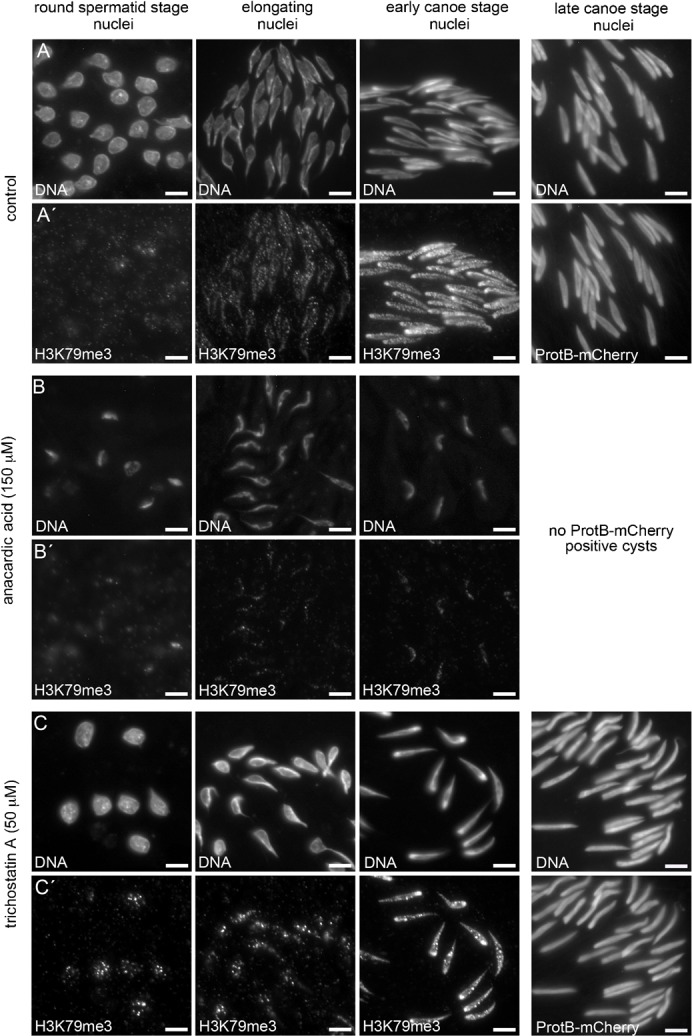
Post-meiotic H3K79 methylation largely depends on histone acetylation. Squashed preparations of spermatid nuclei derived from cultured pupal testes of *protB*-*mcherry* flies incubated for 24 h with either detergent (control; A,A′), the acetyltransferase inhibitor anacardic acid (B,B′), or the histone deacetylase inhibitor trichostatin A (C,C′). (A′,B′,C′) H3K79 methylation was detected by anti-H3K79me3 staining. (A,B,C) DNA was visualized by Hoechst staining. After treatment with anacardic acid, H3K79me3 was severely reduced in spermatids displaying severely compacted chromatin (B′, columns 1–3) compared to control samples (A′), and no protB-mCherry-positive spermatids were present (B,B′; column 4). Treatment with trichostatin A did not lead to premature or aberrant distribution of H3K79me3 (C′). Scale bars: 5 µm.

We blocked histone acetylation by treating cultured pupal testes with anacardic acid, a well known inhibitor of histone acetyltransferases. In agreement with our previous report, no histone H4 hyperacetylation was detected in spermatid nuclei treated with anacardic acid (Awe and Renkawitz-Pohl, 2010). As a consequence, no protB-mCherry-positive cysts were present after 24 h incubation with anacardic acid in contrast to untreated control testes (compare Fig. 5A′ to Fig. 5B′). We asked whether this lack of histone acetylation influences H3K79 methylation. Interestingly, H3K79 methylation detected by anti-H3K79me3 staining of anacardic-acid-treated squashed spermatid nuclei from testes was severely reduced in postmeiotic spermatid nuclei (Fig. 5B′, columns 1–3), and no protamine-positive spermatids were present. The strong reduction of both histone acetylation and H3K79 methylation led to easily recognizable compacted chromatin (compare Fig. 5B to Fig. 5A). We tested whether treatment with the acetyltransferase inhibitor anacardic acid exerts an effect on the methyltransferase Gpp. Importantly, our anti-Gpp antibody allowed us to show that Gpp was not detectable in most cysts at the early canoe stage after anacardic acid treatment (compare Fig. 4B′ to Fig. 4C′). We concluded that the synthesis or nuclear localization of Gpp and the corresponding H3K79 methylation depend – at least partially – on H4 hyperacetylation.

Next, we wanted to know whether premature histone acetylation is sufficient to induce H3K79 methylation. Induction of premature histone acetylation in round spermatids by treatment with the histone deacetylase inhibitor trichostatin A did not lead to severe H3K79 methylation already in round spermatid nuclei (Fig. 5C′), and did not lead to obvious alterations in chromatin remodeling, as recognized by the presence of protB-mCherry spermatids (Fig. 5C′, column 4). These results indicated that H3K79 methylation is directly or indirectly dependent on histone acetylation and importantly also indicated that histone acetylation is not the sole requirement for successful H3K79 methylation.

### Also in rat spermiogenesis, H3K79 methylation precedes histone displacement

**Fig. 6.**
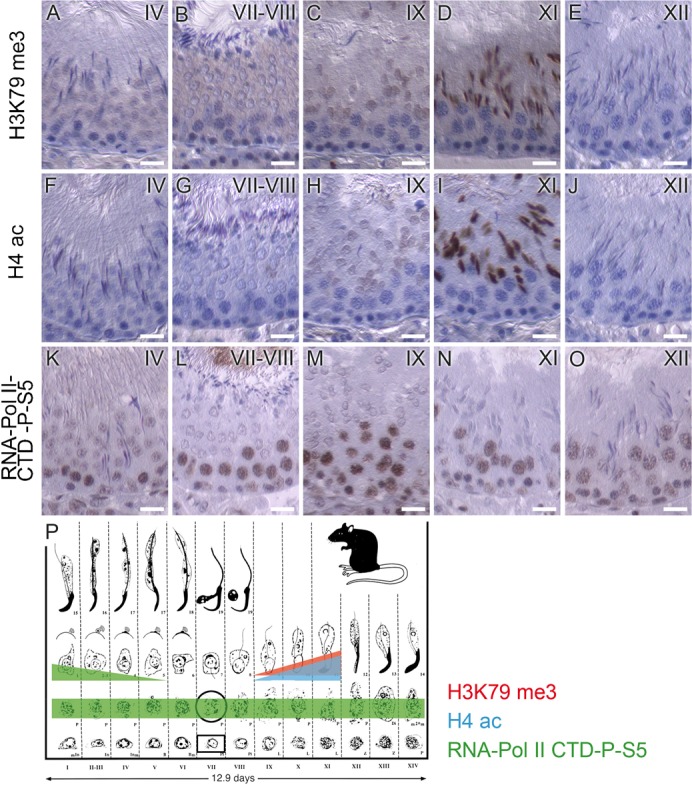
H3K79me3 and H4ac are present in elongating spermatids in rat testes, but localization does not overlap with active RNA polymerase II. (A–E) Sections of rat testes probed with anti-H3K79me3, (F–J) anti-H4ac antibody recognizing highly acetylated histone H4, or (K–O) anti-RNA Pol II-CTD-phosphoS5 antibody recognizing active RNA polymerase II. Both H3K79me3 and H4ac were first detected in spermatids of stage IX tubules (C,H) and were predominantly found in elongating spermatids in stage X–XI tubules (D,I). In contrast, active RNA polymerase II was detected in the nuclei of all primary spermatocytes and in decreasing intensity in round spermatids up to stage IV tubules (K–O). (P) Schematic summary of findings; scheme modified after Russell et al. (Russell et al., 1990). Scale bars: 20 µm.

We next asked whether H3K79 methylation in postmeiotic spermatids is conserved between *Drosophila* and mammals and used testes sections from rat as a model system. We used the anti H3K79me3 antibody, which likely also recognizes di-methylation of H3K79 (see Materials and Methods). We specifically detected H3K79 methylation in elongating spermatids from stage IX tubules (Fig. 6C) onwards until stage XI (Fig. 6D), with the strongest signal for H3K79 methylation present in stages X and XI tubules (spermatogenic staging according to Russell et al., 1990). Of note, the distribution of H3K79 methylation correlated well with the appearance of highly acetylated histone H4 in elongating spermatids in rat (Fig. 6H,I). In mammals, in contrast to *Drosophila*, significant transcription takes place in the haploid phase in round spermatids (Barckmann et al., 2013; Hecht et al., 1986; Kleene, 2003; Rathke et al., 2014). The round spermatid stage lasts for 9 days in rat, and so far it is not clear whether transcription takes place continuously until the elongating spermatid stage. As both modifications – H3K79 methylation and H4 acetylation – are associated with gene expression (Howe et al., 1999; Steger et al., 2008; Vakoc et al., 2006), we compared their distribution with that of active RNA polymerase II. We detected active RNA polymerase II in the nuclei of all primary spermatocytes (Fig. 6K–O) and mainly in round spermatids in tubules at stages I to IV but not in elongating spermatids; H3K79 methylation (Fig. 6C,D) and H4 hyperacetylation (Fig. 6H,I) were visible in elongating spermatids in tubules from stages IX to XI. Consequently, there was little to no overlap between active RNA polymerase II and the investigated histone modifications (see Fig. 6P for summarizing scheme).

Thus, we observed in both *Drosophila* and in rat that both H4 hyperacetylation and H3K79 methylation characterize the histones shortly before they are largely replaced by transition proteins and later by protamines.

## DISCUSSION

We searched for unknown conserved features in chromatin reorganization crucial for the generation of mature fertile sperm in *Drosophila* and rat. We identified H3K79 methylation as a conserved histone modification preceding temporally the process of histone-to-protamine transition. We observed a striking lack of H3K79 methylation before meiotic divisions in both *Drosophila* and rat and comparable distribution of H3K79 methylation in post-meiotic spermatids. The spermatids were characterized by major morphological changes of the nuclei and the initiation of chromatin remodeling, in which histones are replaced by much more smaller DNA-packaging proteins. This methylation of histone H3K79 is generally catalyzed by members of the evolutionary conserved Dot1 family of methyltransferases, which differ from other methyltransferases in the lack of the classical Set1 domain but contain a catalytic active methyltransferase fold (Min et al., 2003; Mohan et al., 2010; Ng et al., 2002). In *Drosophila*, the H3K79 methyltransferase Gpp is known to mediate H3K79 methylation (Shanower et al., 2005). Gpp was mainly found in the nucleolus of spermatocytes and expressed in canoe stage spermatids. The transcript encoding the Gpp-D isoform was present in spermatids. This spermiogenesis-relevant isoform has a unique C-terminus, and this Gpp-D domain might provide binding sites for proteins that regulate the spatiotemporal function of Gpp in the testis in chromatin remodeling during spermiogenesis.

While loss-of-function mutants are embryonic lethal, hypomorphic *gpp*-mutant males are able to reach adulthood. These males show reduced fertility, which suggests that Gpp is indeed required for spermiogenesis. Further studies are required to resolve the network in which Gpp acts during the unique process of chromatin remodeling in spermiogenesis.

Importantly, in both *Drosophila* and rat, the maximum level of H3K79 methylation correlated well with histone H4 hyperacetylation. Highly acetylated histone H4 is associated with histone displacement in mammals (for reviews, see Braun, 2001; Meistrich et al., 1992) and *Drosophila* (Rathke et al., 2007) and is essential for the progression of histone-to-protamine transition in *Drosophila* (Awe and Renkawitz-Pohl, 2010). Here, we showed that H3K79 methylation in post-meiotic spermatids largely depends on the acetylation status of male germ cells. However, we know that another methylation (H3R4) is not affected by anacardic acid (Awe and Renkawitz-Pohl, 2010). The experiments described herein do not allow discrimination between a direct or indirect influence of H4 hyperacetylation on the methylation of histone H3K79. A so-called *trans*-histone crosstalk, in which efficient methylation of lysine H3K79 directly depends on ubiquitinated histone H2B is known to regulate chromatin dynamics during transcription and telomeric silencing in yeast and *Drosophila* (for reviews, see Chandrasekharan et al., 2010; Wood et al., 2005; Mohan et al., 2010). We observed that H3K79 methylation in *Drosophila* is largely blocked if H4 hyperacetylation is inhibited, in agreement with the strongly reduced level of Gpp. Thus, a comparable *trans*-histone crosstalk between H4 acetylation and H3K79 methylation might exist as a mode of regulating chromatin dynamics during spermiogenesis. On the other hand, trichostatin-A-induced premature histone acetylation only led to a minor increase in H3K79 methylation in early spermatids. Taken together, these data might argue for another mechanism in addition to H4 hyperacetylation that regulates Gpp and thus methylation of H3K79 in spermatids. Such a mechanism could also explain our recent finding that premature histone H4 hyperacetylation at the round nuclei stage is not sufficient to induce premature histone displacement (Awe and Renkawitz-Pohl, 2010).

The overlapping distribution of H3K79 methylation and H4 hyperacetylation in spermatids preceding histone removal as well as the dependency of H3K79 methylation on prior histone acetylation strongly indicated that both histone modifications might act in concert to regulate chromatin remodeling during the histone-to-protamine switch. However, as H3K79 methylation and histone H4 hyperacetylation are indicative for an open chromatin configuration, we cannot exclude that these modifications also act at the level of transcription, in particular in spermatocytes. The functional significance of the nucleolar staining of Gpp needs to be clarified in relation to existing data (Chen et al., 2005; El-Sharnouby et al., 2013). Here we focus on the post-meiotic role of Gpp.

In *Drosophila*, in accordance with the low level of post-meiotic transcription, active RNA polymerase II can only be detected for a short time in late canoe stage nuclei (Barreau et al., 2008; Rathke et al., 2007; Vibranovski et al., 2010), a stage in which we hardly observed H3K79 methylation. This argues against a major role of H3K79 methylation in transcription during spermiogenesis in *Drosophila*. In mammals, transcription ceases in mid-spermiogenesis, with high levels of RNA found in haploid round spermatids (Hecht et al., 1986; Kleene et al., 1983). Concordantly, we detected active RNA polymerase II in the nuclei of spermatocytes and early round spermatids in rat testes, whereas H3K79 methylation and H4 hyperacetylation were only detectable in elongating spermatids, which are devoid of actively transcribing RNA polymerase II. Based on these observations in *Drosophila* and rat, we propose that H3K79 methylation and H4 acetylation act together – directly or indirectly – in opening the chromatin structure to facilitate access of regulatory proteins needed for histone replacement rather than in regulating gene expression.

In summary, we identified H3K79 methylation as a conserved histone modification in spermatids. In *Drosophila*, a single gene, *gpp* (*grappa*), encodes a Dot1l-like H3K79 methyltransferase. In spermatids, Gpp expression corresponds to the H3K79 methylation pattern, and both depend on previous histone acetylation. It remains to be elucidated whether and how these histone modifications influence the structure of the chromatin to progress to or allow histone removal and the stepwise deposition of DNA-packaging proteins, such as transition proteins and protamines.

## Supplementary Material

Supplementary Material
