# Supplementary material for: H3K79 methylation: a new conserved mark that accompanies H4 hyperacetylation prior to histone-to-protamine transition in Drosophila and rat
Source: Biol Open. 2014 May 2;3(6):444–52. doi: 10.1242/bio.20147302 (PMC4058078; doi:10.1242/bio.20147302)
Supplement: Supplementary Material [file supp_3_6_444__index.html]

H3K79 methylation: a new conserved mark that accompanies H4 hyperacetylation prior to histone-to-protamine transition in Drosophila and rat — H3K79 methylation: a new conserved mark that accompanies H4 hyperacetylation prior to histone-to-protamine transition in Drosophila and rat — Supplementary Material 

# H3K79 methylation: a new conserved mark that accompanies H4 hyperacetylation prior to histone-to-protamine transition in *Drosophila* and rat

## bio.20147302 Supplementary Material

**Files in this Data Supplement:**

- Supplementary Material - Christine Dottermusch-Heidel et al. doi: 10.1242/bio.20147302
