# Supplementary material for: H3K79 methylation: a new conserved mark that accompanies H4 hyperacetylation prior to histone-to-protamine transition in Drosophila and rat
Source: Biol Open. 2014 May 2;3(6):444–52. doi: 10.1242/bio.20147302 (PMC4058078; doi:10.1242/bio.20147302)
Supplement: Supplementary Material [file supp_bio.20147302_bio.20147302-s1.pdf]

Biology Open (2014) 000, 1–9 doi:10.1242/bio.20147302

Supplementary Material

Christine Dottermusch-Heidel et al. doi: 10.1242/bio.20147302

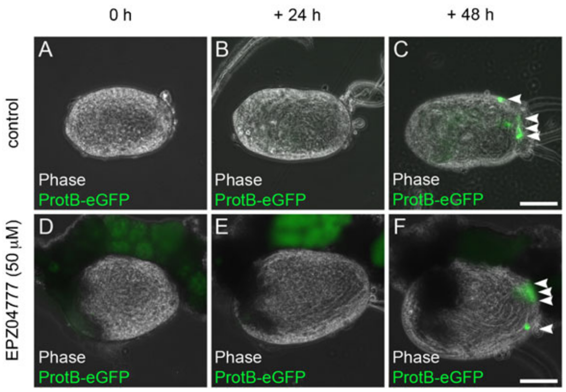

Fig. S1. Treatment with Dot1l inhibitor EPZ04777 does not inhibit the histone-to-protamine switch in cultured intact testes of *Drosophila*. Pupal testes [0 h after puparium formation (APF)] of *Drosophila* expressing ProtB-eGFP were incubated for 48 h (A–C) in medium with detergent (DMSO) as control or (D–F) in medium supplemented with the Dot1l inhibitor EPZ04777 (50 µM). No ProtB-eGFP positive cysts were observed at the beginning of culture and after 24 h of incubation in both the control (A,B) and in inhibitor-treated cultures (D,E). After 48 h of incubation, some cysts have undergone the histone-to-protamine switch, as seen by the ProtB-eGFP-positive cysts (C,F, arrowheads). The numbers of ProtB-eGFP-expressing cysts in the control and inhibitor-treated cultures were similar. Scale bars: 100 µm.

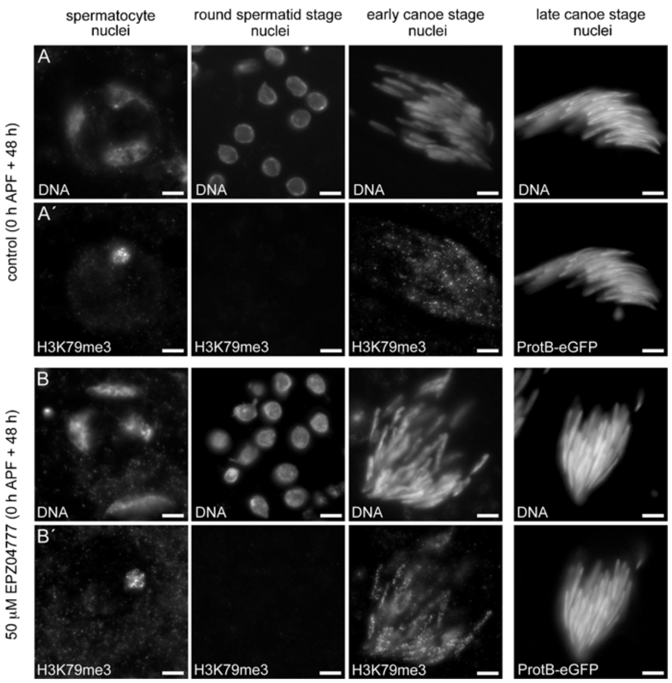

Fig. S2. Treatment with Dot1l inhibitor EPZ00477 does not inhibit methylation of histone H3K79 and ProtB-eGFP expression. Squash preparations of spermatid nuclei derived from cultured pupal testes of *Drosophila* expressing protB-eGFP [0 h after puparium formation (APF)] incubated (A,A′) with detergent (DMSO) as control or (B,B′) with the Dot1l inhibitor EPZ04777 (50 µM) for 48 h. (A′,B′, columns 1–3) H3K79 was detected by anti-H3K79me3 staining. (A,B) DNA was visualized by Hoechst staining. (A′,B′, column 4) ProtamineB expression was visualized by the ProtB-eGFP fusion protein at the late canoe stage. No changes in H3K79me3 distribution or ProtB expression were visible after treatment with EPZ04777. Scale bars: 5 µm.

Biology Open (2014) 000, 1–9 doi:10.1242/bio.20147302

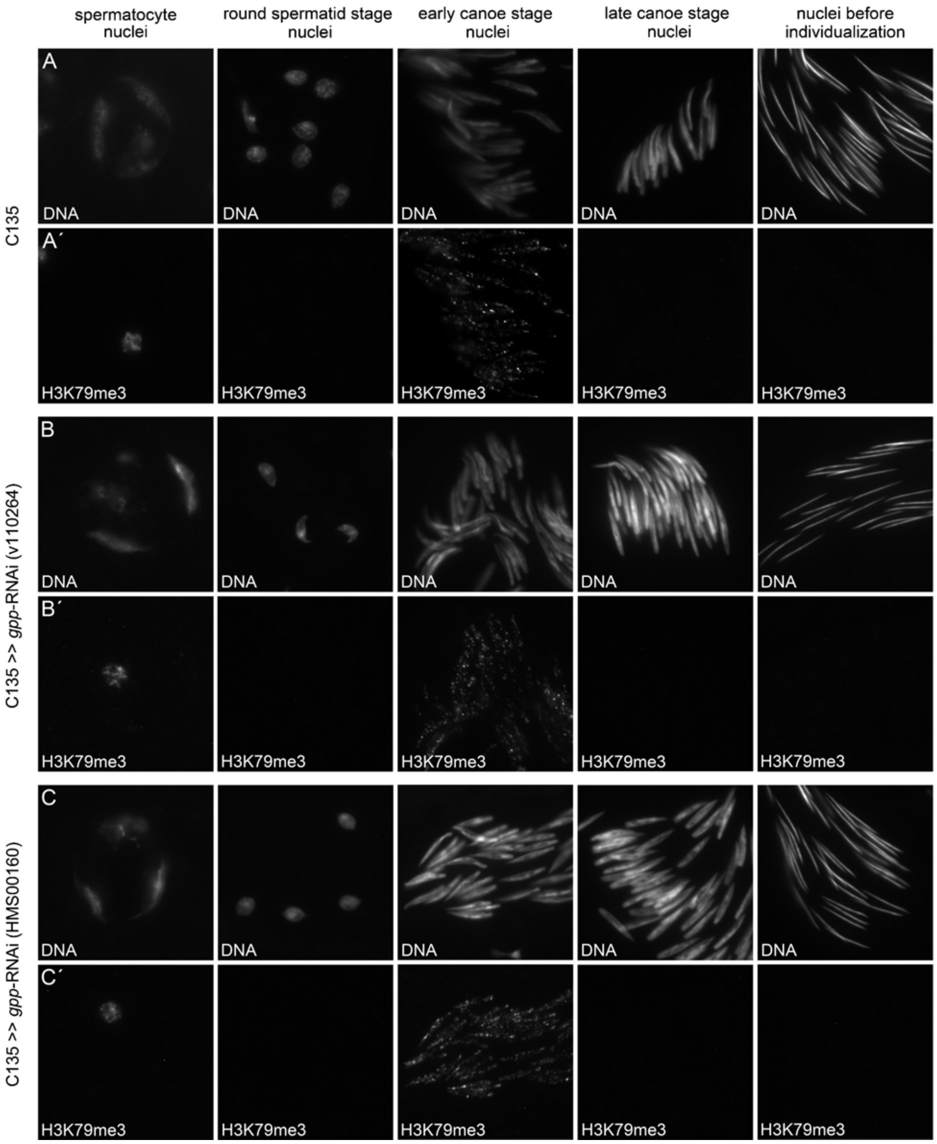

Fig. S3. RNAi directed against *gpp* does not inhibit H3K79me3 in postmeiotic spermatids. Squash preparations of spermatid nuclei derived (A,A′) from *Drosophila* harboring the c135-Gal4 driver alone and (B,B′) from *Drosophila* expressing the gpp-RNAi construct v110264 or (C,C′) HMS00160 under control of c135 in spermatocytes. (A′,B′,C′) H3K79 methylation was detected by anti-H3K79me3 staining. (A,B,C) DNA was visualized by Hoechst staining. No changes in H3K79me3 distribution in early canoe stage spermatids were detected after expression of gpp-RNAi under control of C135-Gal4 (B′,C′) in comparison to the control (A′).

Biology Open

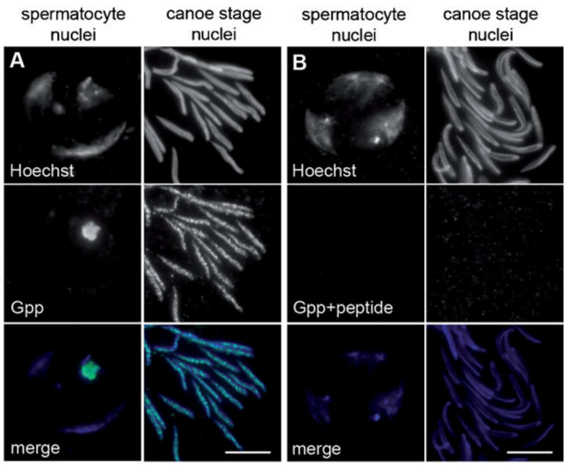

Fig. S4. Anti-Gpp antibody blocking with immunizing peptide. (A) Anti-Gpp staining of squashed spermatid nuclei from testes of wild-type *Drosophila*. DNA was visualized by Hoechst staining. Gpp was detected in spermatocyte (mainly in the nucleolus) and canoe stage nuclei. (B) Staining with peptide-neutralized anti-Gpp antibody of squashed spermatid nuclei from testes of wild-type *Drosophila*. DNA was visualized by Hoechst staining. After peptide blocking, Gpp was no longer detected in spermatocyte and canoe stage nuclei. Scale bars: 10 µm.

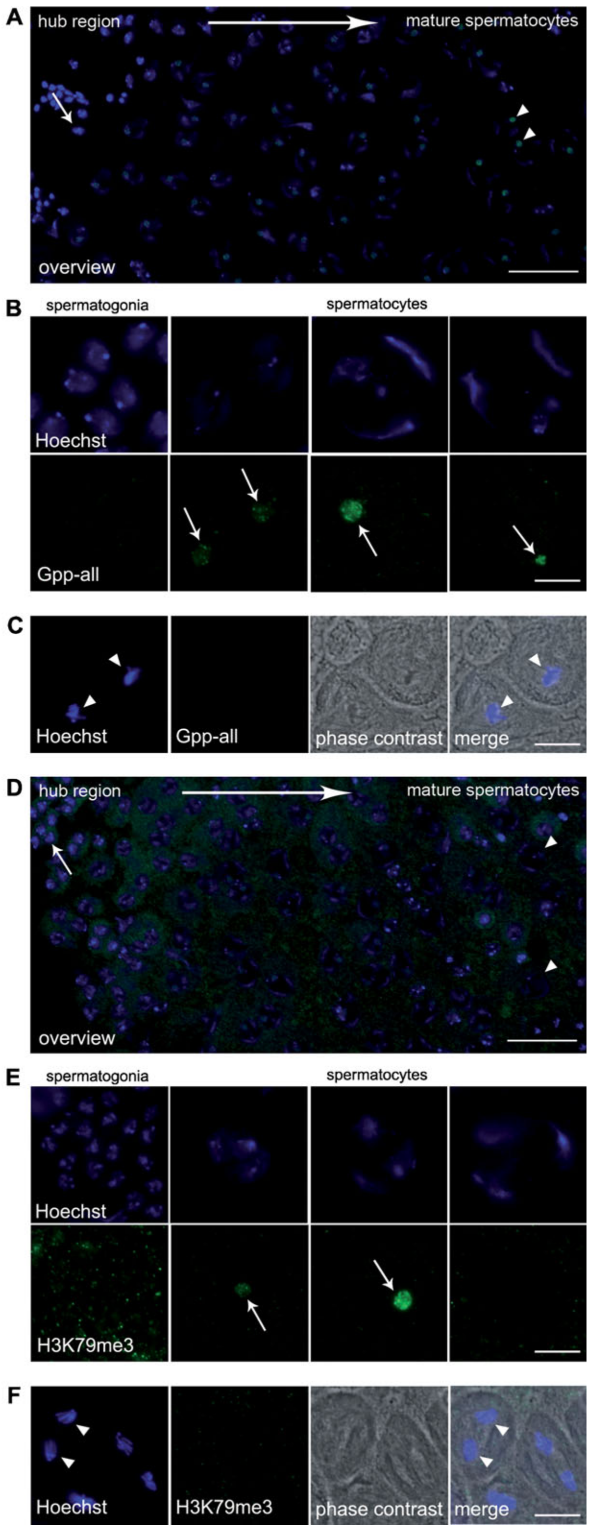

Fig. S5. Gpp and H3K79me3 are hardly detectable before and during meiotic division. (A,D) Overviews of a larval testes, with the hub with adhering stem cells oriented toward the left, spermatogonia marked by an arrow, and mature spermatocytes (arrowheads) shortly before meiotic division or in division oriented toward the right. (A) Merged image of Hoechst and anti-Gpp staining. (D) Merged image of Hoechst and anti-H3K79me3 staining. (B,E) Spermatogonia and nuclei of three subsequent spermatocyte stages; nucleoli are marked with an arrow. (C,F) Meiotic divisions with Hoechst staining, anti-H3K79me3 staining, and phase contrast, as indicated. Arrowheads mark the chromosomes. Scale bars: 10 µm (B,C,E,F), 50 µm (A,D).
